# Screening and Perioperative Management of Obesity Hypoventilation Syndrome

**DOI:** 10.3390/jcm13175000

**Published:** 2024-08-23

**Authors:** Roop Kaw, Kara Dupuy-McCauley, Jean Wong

**Affiliations:** 1 Department of Hospital Medicine, Outcomes Research Consortium, Cleveland Clinic, Cleveland, OH 44195, USA; 2 Department of Pulmonary, Critical Care and Sleep Medicine, Mayo Clinic, Rochester, MN 55905, USA; dupuy-mccauley.kara@mayo.edu; 3 Department of Anesthesiology and Pain Medicine, Toronto Western Hospital, University Health Network, Toronto, ON M5T 2S8, Canada; jean.wong@uhn.ca

**Keywords:** obesity hypoventilation syndrome, perioperative management, postoperative respiratory failure

## Abstract

Obesity hypoventilation syndrome (OHS) can often be underdiagnosed or misdiagnosed and has been shown to pose significant risks in perioperative situations. Patients with OHS have a higher prevalence of baseline morbid conditions like hypertension, congestive heart failure (CHF), diabetes mellitus, atrial fibrillation, and pulmonary hypertension (PH), which contribute to adverse postoperative outcomes. The potential challenges include difficult intubation and loss of airway, postoperative respiratory failure, worsening heart failure, pulmonary hypertensive crisis, and opioid-induced respiratory depression (OIRD). It is, therefore, important to screen all obese patients for obstructive sleep apnea (OSA) and OHS before elective surgical procedures. The aim of this review is to discuss the preoperative screening and evaluation and safe anesthetic and up-to-date ventilatory management of this complex group of patients. This review also intends to increase the awareness of OHS in the adult population among hospitalists, surgeons, and cardiologists who may find themselves taking care of these patients in complex multidisciplinary settings.

## 1. Introduction

The current definition of OHS includes obesity (BMI of ≥30 kg/m^2^), the presence of sleep-disordered breathing (apnea–hypopnea index (AHI) ≥ 5 in 90% of cases, AHI < 5 in 10% of cases), and awake alveolar hypoventilation with a daytime PaCO_2_ ≥ 45 mmHg in the absence of other causes of hypoventilation. The development of OHS can be described in five stages. Stage 0 is the presence of OSA alone without hypoventilation. Stages I and II include the presence of obesity-related hypoventilation during sleep only, and stages III and IV involve daytime hypercapnia and would warrant a diagnosis of OHS [1]. Sleep-related hypoventilation (SRH) is considered a separate diagnosis and can occur in many conditions of hypoventilation; for example, chronic obstructive pulmonary disease (COPD) or neuromuscular disease. Due to the drop in minute ventilation that occurs during sleep even in healthy subjects, SRH is often a precursor to daytime hypercapnia.

The prevalence of OHS in patients with OSA ranges from 9 to 38% based on reports in the current literature, and prevalence increases with a higher BMI [2,3]. The prevalence of OHS in the general population has not been directly assessed, but extrapolated measurements based on the prevalence of severe obesity (BMI ≥ 40 kg/m^2^) and OSA suggest that the prevalence of OHS in the general United States population is approximately 0.15–0.3% [4]. The prevalence of OHS may be higher in men; however, there are conflicting data in the literature regarding the ratio of men to women affected. There is also no clear racial or ethnic predominance, independent of BMI. People with OHS have higher healthcare utilization in the years preceding and after diagnosis [5] and a lower quality of life [6]. This is likely due at least in part to the exceedingly high prevalence of co-morbidities in patients with OHS.

## 2. Clinical Presentation and Preoperative Evaluation

Patients with OHS may have signs and symptoms of excessive daytime sleepiness, fatigue, loud snoring, witnessed apneas, nocturia, dyspnea, nocturnal hypoxemia, and mild daytime hypoxemia. Unfortunately, OHS is more commonly first recognized during hospital admission for respiratory failure as opposed to the outpatient setting [7,8,9,10]. But, recognition in the hospital is still poor, as only 13% of patients who meet OHS criteria are discharged from the hospital with an appropriate diagnosis and treatment plan [8]. The prevalence of OHS in hospitalized patients with BMI > 35 kg/m^2^ has been reported to be as high as 31% [8], but OHS often either goes unrecognized or is mistaken for another condition associated with hypercapnic respiratory failure, such as COPD or congestive heart failure (CHF) [11]. During outpatient evaluation, a reduction in forced vital capacity (FVC) as part of pulmonary function testing [4,12] and daytime oxygen saturation ≤ 95% are both predictive of OHS in high-risk patients with OSA [13].

### 2.1. Polysomnography

**Figure 1:**
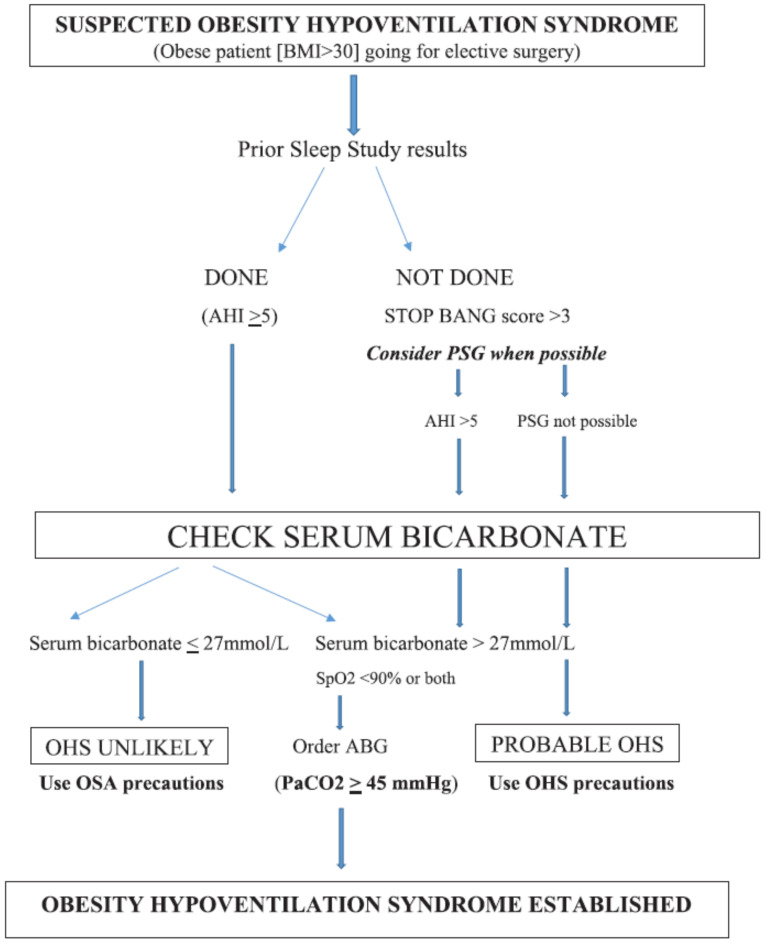
Perioperative decision tree in patients with suspected OHS (with permission from (https://journals.lww.com/anesthesia-analgesia/fulltext/2021/05000/obesity_and_obesity_hypoventilation,_sleep.13.aspx, accessed on 21 August 2024)).

Although it is not practical to obtain polysomnography (PSG) before elective non-cardiac surgery (NCS), the Society of Anesthesia and Sleep Medicine recommends additional preoperative evaluation in patients with OSA in the presence of the following: 1. hypoventilation, 2. pulmonary hypertension, and 3. resting hypoxemia not attributable to cardiopulmonary diseases [14]. Therefore, when possible, consideration should be given to obtaining PSG preoperatively (Figure 1) while keeping in mind that no evidence exists to delay or cancel the proposed elective surgery in such situations. PSG with carbon dioxide monitoring is the gold standard for diagnosing SRH. The most recent diagnostic criteria from the American Academy of Sleep Medicine for hypoventilation during sleep includes PCO_2_ > 55 mmHg for > 10 min during sleep or an increase in PCO_2_ ≥ 10 mmHg as compared to the awake supine value up to a level of > 50 mmHg for ≥10 min [15], and this finding should be present for both patients with OHS and patients with isolated SRH.

### 2.2. Serum Bicarbonate and Arterial Blood Gas Analysis

In addition to establishing the presence of sleep-disordered breathing, the patient must have an arterial blood gas (ABG) measurement to establish the presence of awake hypercapnia. Serum bicarbonate can act as a surrogate for hypercapnia when elevated in the absence of other plausible causes and can function as a screening tool for some patients with OHS. The American Thoracic Society practice guideline recommendation states that for patients with a moderate–high probability of having OHS (20%, usually based on BMI > 40 kg/m^2^), a serum bicarbonate level of <27 mmol/L precludes the need for ABG with a 99% negative predictive value. A serum bicarbonate level of ≥27 mmol/L in this same patient population (20% prevalence of OHS) provides a 48.3% positive predictive value for OHS and should prompt evaluation with an ABG [16].

Another workup when, and as possible preoperatively, centers around other causes of hypoventilation and may include pulmonary function testing, chest imaging, an electrocardiogram, or echocardiography, and, especially if PH is suspected, thyroid function tests and neurological evaluation. Importantly, finding a lower FVC on PFT at the time of diagnosis can be correlated to the risk of ICU admission for acute hypercapnic respiratory failure, so patients with a low FVC may warrant additional assessment [17]. 

## 3. Pathophysiology That Can Play into Perioperative Complications and Consequent Management

Respiratory mechanics are impaired in patients with obesity. Patients with obesity may have atelectasis without adjustment of hypoxic pulmonary vasoconstriction, resulting in alveolar–arterial gradient hypoxemia and ventilation/perfusion mismatch [18]. Obesity affects lung volumes, typically with reduced expiratory reserve volume (ERV), decreased functional residual capacity (FRC), and sometimes decreased total lung capacity [19]. A reduced FRC and ERV leads to rapid shallow breathing with smaller tidal volume and increased dead space ventilation [20]. The mechanical load on the diaphragm and chest wall from adipose tissue decreases chest wall compliance, which is further reduced in the supine position [21]. The rapid shallow breathing and decreased chest wall compliance lead to increased work of breathing with increased resting energy expenditure [22]. Due to this increased work of breathing, patients with obesity need a higher central respiratory drive to maintain levels of oxygen and carbon dioxide, but patients with OHS do not maintain eucapnia due to a blunted central respiratory drive, which may be due to leptin resistance. Adipose tissue is endocrinologically active and produces hundreds of adipocytes. Leptin is an adipokine, which is a respiratory stimulant and is thought to play a role in ventilatory control. Leptin is elevated in patients with obesity, but hyperleptinemia in OHS is associated with reduced respiratory drive and blunted hypercapnic response [23] due to central leptin resistance, and it is evident that reduced central chemosensitivity resulting in chronic hypercapnia increases the effects on the respiratory system beyond what we see in patients with eucapnic obesity and eucapnic OSA. When compared to patients with eucapnic OSA, patients with OHS have more severe upper airway obstruction [24], increased work of breathing [25], impaired respiratory mechanics [26], and blunted central response to hypoxemia and hypercapnia [27]. These factors lead to decreased respiratory reserve and increased risk of respiratory failure.

Obesity has also been associated with PH [28], likely, at least in part, due to the alterations in left ventricular function [29,30], high cardiac output due to increased blood volume supplying excess adipose tissue [31], and fatty infiltration of the myocardium, leading to cardiomyopathy [32]. OHS carries a much higher risk of PH, which is likely conferred by chronic hypercapnia and nocturnal hypoxemia [33]. Unrecognized associated PH at the time of surgery becomes another risk factor for perioperative complications.

The increased risk of respiratory failure is an important consideration for patients with OHS during hospitalization, including patients who are undergoing elective surgery. During hospitalization, people with OHS had higher rates of ICU admission and the need for invasive mechanical ventilation when compared to people with simple obesity without chronic hypercapnia [8]. Early research suggested an increased rate of mortality of up to 50% for hospitalized patients with OHS [34,35], although this finding has not been confirmed in more recent studies [8,36]. Medications commonly administered peri-operatively, including many anesthetics and analgesics, can reduce respiratory drive, increase arousal threshold, and worsen sleep-disordered breathing, so patients with these problems at baseline are particularly vulnerable to their effects [37]. Compared to OSA without daytime hypercapnia, patients with OHS have been shown to have an increased risk of respiratory failure, heart failure, prolonged intubation, tracheostomy, and ICU transfer [38] and a higher length of stay both in the ICU and the hospital. This may suggest that there is a role for screening patients for OHS prior to ambulatory surgery, although no current guidelines for preoperative OHS assessment exist and there are many knowledge gaps in the current literature [39].

## 4. OHS Patient and Ambulatory Surgery

The suitability of performing ambulatory surgery on patients with OHS should be carefully considered based on the invasiveness of the procedure, co-morbidities, adherence to positive airway pressure therapy, and postoperative opioid requirements [14]. Unlike ambulatory surgery performed at hospitals, office-based practices or free-standing ambulatory surgical centers may not have adequate airway equipment, staffing, postoperative monitoring capabilities, and the ability to admit patients for perioperative complications. A full summary of needs assessment and recommendations is provided in Table 1.

## 5. Intraoperative Considerations

### 5.1. Airway Management

As patients with OHS are at higher risk for difficult mask ventilation and tracheal intubation, the availability of difficult airway equipment is of paramount importance. Video laryngoscopy, supraglottic airway devices, and high-flow or low-flow oxygen should be available. However, if difficult mask ventilation is suspected, awake bronchoscopic intubation with local anesthesia may be considered.

### 5.2. Positioning and Pre-Oxygenation

Patients with OHS should be positioned in a head elevation laryngoscopy position (HELP) or ramped position during pre-oxygenation and induction of anesthesia. This position may be achieved by tilting the operating table head up, placing blankets underneath the patient’s head and shoulders, or using an elevation pillow to obtain a horizontal alignment of the external auditory meatus with the sternal notch. An elevation pillow, such as the Troop Elevation Pillow, places patients with morbid obesity in an ideal position for both pre-oxygenation and intubation [40]. This position results in proper alignment of the oral, pharyngeal, and laryngeal axes in morbidly obese patients, improving the glottic view during laryngoscopy. Randomized studies show that the ramped position significantly improves the laryngeal view, reduces intubation time, and lowers the incidence of difficult mask ventilation of morbidly obese patients compared to the sniffing position [41,42]. Pre-oxygenation of the patient in a 25-degree head-up position, as opposed to the supine position, resulted in higher PaO_2_ levels and prolonged the duration of time to desaturate to 92% during an apnea [43]. Highly trained personnel, such as anesthesiology assistants or respiratory therapists, should also be readily available to assist with airway management.

Apneic oxygenation with nasal cannula during laryngoscopy prolongs the time to oxygen desaturation in obese patients with suspected difficult airways [44]. In morbidly obese patients, high-flow nasal oxygenation compared with oxygenation via a face mask during the induction of anesthesia increased the time before desaturation [45,46]. If high-flow nasal oxygen is not available, low-flow nasal oxygen has also been shown to prolong the time to desaturation during prolonged laryngoscopy in obese surgical patients [44,47]. Also, continuous positive airway pressure (CPAP) of up to 10 cm H_2_O for 5 min until the time of intubation can reduce the decrease in FRC due to a change in body position, muscle relaxation, and absorptive atelectasis from the high inspired concentration of oxygen, thereby increasing safe apnea time [48].

### 5.3. Type of Anesthesia

#### 5.3.1. Local/Regional Anesthesia

While there is limited literature on patients with OHS undergoing surgery, for patients with OSA, the American Society of Anesthesiologists and the Society of Anesthesia and Sleep Medicine strongly recommend local or regional anesthesia techniques rather than general anesthesia when appropriate [49,50]. Local or regional anesthesia techniques can mitigate airway complications and respiratory depression associated with opioids and sedation from general anesthesia and residual neuromuscular blockade. Given that patients with OHS already have diminished respiratory drive, opting for regional anesthesia over general anesthesia and airway manipulation is preferable [27]. However, in morbidly obese individuals, regional anesthesia techniques can be challenging, necessitating specialized equipment, such as longer spinal or peripheral nerve block needles, as well as ultrasound or fluoroscopic guidance [51]. It is important to consider that obese patients may not tolerate supine or Trendelenburg positioning during the surgical procedure while under regional anesthesia. Local or regional anesthesia techniques also reduce postoperative pain, the need for opioids, and postoperative nausea and vomiting. Intrathecal opioids should be avoided due to concerns of delayed respiratory depression.

Minimal sedation should be administered during surgery performed with local or regional anesthesia in order to prevent airway obstruction, hypoventilation, and hypercapnia. When sedation is given, continuous capnography is recommended to promptly detect any signs of airway obstruction. Additionally, bringing the patient’s positive airway pressure (PAP) device into the operating room may be considered, especially if moderate sedation is planned.

#### 5.3.2. General Anesthesia

When general anesthesia is necessary, short-acting drugs at the lowest doses possible should be utilized. Unfortunately, the appropriate dosing of anesthetic agents is unclear, as patients with morbid obesity have often been excluded from clinical trials during the drug development phase [52]. Although it has been recommended to dose patients with morbid obesity based on lean body weight (LBW), there is no consensus on which dosing scalar to use. Patients with morbid obesity have increased cardiac output, lean body weight, fat mass, volume of distribution, and changes in regional blood flow, which affect the pharmacokinetics and pharmacodynamics of anesthetic drugs [53,54,55]. Increased cardiac output in obese individuals leads to more rapid onset and offset of drug effects, lower peak plasma concentrations and a shorter duration of effect of a bolus dose of the drug. The lipophilicity and volume of distribution are important to consider when dosing anesthetic drugs [56]. For bolus or loading doses, the volume of distribution of lipophilic drugs is greater, whereas the volume of distribution of less lipophilic drugs is relatively unchanged compared to normal-weight individuals. Prolonged infusions of lipophilic drugs, such as propofol, may lead to delayed recovery due to the sequestration of lipophilic drugs in poorly vascularized adipose tissue [57]. Anesthetic medications should be titrated to clinical effects during induction and maintenance. The use of depth of anesthesia monitoring has been suggested to help guide the administration of anesthesia in morbidly obese patients [58,59]. Short-acting volatile anesthetics, such as sevoflurane and desflurane, will allow more rapid recovery at the end of surgery, as patients with OHS have blunted hypoxic and hypercapnic drives, increasing the risk for postoperative hypoxemia [60].

Multi-modal non-opioid analgesia is recommended intraoperatively to minimize opioid administration [50]. Patients with OHS have diminished respiratory drive, and this may worsen postoperatively. Short-acting opioids, such as remifentanil or fentanyl, should be used rather than longer-acting opioids. Using remifentanil intraoperatively may facilitate using lower doses of volatile anesthesia and lead to more rapid recovery [61]. Local anesthesia infiltration or peripheral nerve blocks with long-acting local anesthesia will also help reduce pain and the need for opioids. Dexamethasone 8 mg/kg intravenous is often given as part of anti-emetic prophylaxis but also modestly reduces postoperative pain and opioid consumption, particularly after orthopedic, oral, ear, nose, and throat surgery [62]. A single dose of dexamethasone did not increase the risks of adverse events, such as surgical site infection [62]. 

Non-depolarizing neuromuscular blocking agents are hydrophilic compounds that have a small volume of distribution and undergo hepatic and renal metabolism. These agents should be dosed using LBW. In contrast, obese patients have increased levels of plasma pseudo-cholinesterase, which metabolizes succinylcholine; therefore, dosing of succinylcholine should be based on TBW [63]. Continuous quantitative neuromuscular monitoring is recommended to measure the level of neuromuscular blockade intraoperatively in order to minimize the dose of neuromuscular blocking agent administration and ensure adequate reversal at the time of extubation [64]. The dosing of neostigmine should be based on TBW, but the dosing of sugammadex is controversial as to whether TBW, LBW, or ideal body weight should be used; nevertheless, higher dosing results in a faster reversal time [65].

### 5.4. Intraoperative Ventilation Strategies

Intraoperative protective mechanical ventilation strategies, such as low tidal volumes (TV) of 6 to 8 mL/kg of predicted body weight, low levels of positive end-expiratory pressure (PEEP), and lung recruitment in the form of 30 s of continuous positive airway pressure at 30 cm H_2_O, decrease the incidence of postoperative pulmonary and extrapulmonary complications in non-obese patients undergoing laparoscopic or open abdominal surgery [66]. Nonetheless, whether lung protective ventilation in morbidly obese surgical patients reduces postoperative pulmonary complications is unclear, and patients with OHS have not been included in previous trials on lung protective ventilation. Applying low levels of positive end-expiratory pressure (PEEP) of 5 to 10 cm H_2_O reduced atelectasis after laparoscopic bariatric surgery [67]. Although the optimal level of PEEP for obese patients is unknown, high levels of PEEP (12 cm H_2_O) and alveolar recruitment maneuvers failed to reduce postoperative pulmonary complications in obese patients undergoing abdominal surgery when compared to low levels of PEEP (4 cm H_2_O) [68]. While the high level of PEEP was associated with a reduction in hypoxemia, it also led to a higher incidence of intraoperative hypotension and bradycardia. Consequently, it is advisable to exercise caution with elevated PEEP levels in patients with OHS, given their propensity for co-morbidities and reduced tolerance to hypotension. Although individualized PEEP compared to either fixed low levels (4–5 cm H_2_O) or a higher level (12 cm H_2_O) improved intraoperative oxygenation, driving pressures, and indices of regional ventilation in obese patients undergoing laparoscopic abdominal surgery, there was, again, no difference in pulmonary complications [69]. Pressure control ventilation improves intraoperative oxygenation when compared to volume control ventilation and thus may be an alternative mode of ventilation in patients undergoing laparoscopic surgery [70].

## 6. Postoperative Considerations

### 6.1. Postoperative Complications

As patients with OHS have a higher prevalence of serious co-morbidities, including CHF, CAD, cor pulmonale, and PH, they are at higher risk for postoperative complications, including respiratory failure, prolonged intubation, heart failure, postoperative intensive care unit (ICU) admission, and longer ICU and hospital stay, when compared to those with OSA [71].

### 6.2. Extubation and Postoperative Recovery

Patients with OHS are vulnerable to post-extubation airway obstruction and residual respiratory depression from anesthetic agents. These patients should be extubated only after they are fully awake and have complete reversal of neuromuscular blockade, as evidenced by quantitative monitoring, in the semi-sitting or upright position [49,50]. Residual anesthesia or a neuromuscular blockade (train-of-4 ratio < 0.9) will worsen hypoventilation, resulting in acute respiratory acidosis and further blunting of respiratory drive, which can lead to worsening hypercapnia and hypoxemia when supplemental oxygen is used in patients with OHS [71,72].

Implementing non-invasive ventilation (NIV) immediately after extubation reduces post-extubation respiratory failure and prevents re-intubation in obese patients in the ICU [73]. In patients with OSA, CPAP improves oxygenation and reduces the need for re-intubation and mechanical ventilation after surgery [74]. Postoperatively, for patients with OHS, an empirical approach using bi-level pressure settings, including inspiratory PAP within the range of 18 to 20 cm H_2_O and an expiratory PAP of 8 to 10 cm H_2_O, can be tried based on established norms for the management of patients with OHS. In patients with REM sleep hypoventilation and/or impaired respiratory mechanics, ventilation can be increased by increasing inspiratory positive airway pressure (IPAP) or/and decreasing expiratory positive airway pressure (EPAP), resulting in higher pressure support and TV, decreasing dead space ventilation, and increasing the respiratory rate. Notably, in the early postoperative phase, bi-level PAP ventilation should include a backup respiratory rate (bi-level PAP spontaneous/timed or ST). The backup respiratory rate should be adjusted to two to three breaths per minute lower than the patient’s spontaneous rate prior to surgery while considering that the respiratory rate may be reduced until the patient recovers from anesthetic agents and sedatives. The patient’s CPAP or bi-level PAP should be brought to the post-anesthesia care unit (PACU) for immediate use after the patient leaves the operating room.

Although it is standard practice to administer supplemental oxygen therapy in the PACU, supplemental oxygen may prevent early detection of opioid-induced respiratory depression (OIRD) when just pulse oximetry is used [75]. In addition to PAP, up to 40% of patients with OHS are prescribed nocturnal home oxygen [76]. The prolonged use of high concentrations of supplemental oxygen alone without PAP therapy should be avoided in patients with OHS, as this can worsen hypoventilation and hypercapnia [77,78]. If patients with OHS require high-flow oxygen after surgery, especially while on intravenous patient-controlled analgesia, monitoring should include capnographic monitoring of ventilation [79].

Patients with OHS have a decreased hypoxic ventilatory drive and an increased risk for OIRD. Opioid administration should be minimized and multi-modal non-opioid analgesics, such as acetaminophen and non-steroidal anti-inflammatory drugs or cyclooxygenase (COX-2) inhibitors, may reduce the risk for OIRD. These analgesics can be started preoperatively and continued postoperatively. A large retrospective study found that concomitant use of gabapentinoids with opioids for postoperative analgesia was associated with an increased risk of opioid overdose, respiratory depression, and other opioid-related adverse events [80].

### 6.3. Postoperative Monitoring in the PACU

Monitoring the patient with OHS in the PACU is critical to detect recurrent respiratory events, including apnea, bradypnea, hypercapnia, oxygen desaturation, or pain–sedation mismatch. Patients with recurrent respiratory events in the PACU are at higher risk for postoperative respiratory complications after discharge from the PACU, and these patients will need increased postoperative monitoring [81]. The need for opioids and the ability to resume PAP therapy are important considerations for the postoperative disposition and monitoring requirements. Patients with OHS should be maintained in a semi-sitting or sitting position while in the PACU and on the ward in order to reduce upper airway collapsibility, decrease atelectasis, decrease dead space ventilation, and reduce upper airway edema from intubation and intraoperative fluid administration [74].

### 6.4. Challenges and Future Research

OHS is often underdiagnosed and should be suspected in ambulatory settings in patients with OSA who are desaturated during the day and in hospitalized obese patients complicated by respiratory failure. It is not uncommon for OHS to be diagnosed for the first time in the postoperative setting [38]. There are limited data to guide the management and, more importantly, the monitoring of patients with OHS in the non-ICU setting, and better studies are needed. Patients with OHS have a high prevalence of CHF, particularly heart failure with preserved ejection fraction (HFpEF), which, by itself, can be difficult to diagnose in obese patients [82]. Further research is needed to determine the most effective ventilation strategies for obese surgical patients, including optimal versus individualized PEEP levels, preservation of recruitment maneuvers after tracheal extubation, and limiting inspired oxygen levels. The role of dexmedetomidine, clonidine, intravenous lidocaine, low-dose ketamine, and low-dose magnesium for postoperative analgesia in patients with OHS is another important area for future research.

## Figures and Tables

**Table 1:** Recommendations for the perioperative management of OHS.

- In patients with known or suspected OSA, consider additional preoperative evaluation when there is evidence of hypoventilation, pulmonary hypertension, and/or resting hypoxemia not attributable to other cardiopulmonary diseases.
- Patients with severe OSA should be screened for OHS preoperatively.
- Serum bicarbonate should be part of routine screening for OHS. A relatively recent serum bicarbonate (in the last 2–3 mo) ≤27 mEq/L is very useful to exclude OHS.
- If, in a severely obese patient, the clinical suspicion for OHS is high, consider obtaining an ABG preoperatively if the serum bicarbonate is >27 mEq/L.
- Although there is a paucity of literature on OHS patients, local or regional anesthesia techniques wherever possible avoid airway complications and respiratory depression due to residual neuromuscular blockade and sedation associated with general anesthesia.
- Ramped position is preferred for induction and intubation.
- Avoid sedatives as premedication as much as possible.
- Apneic oxygenation with high-flow nasal oxygen during laryngoscopy may help prevent desaturation, increase safe apnea time during induction of general anesthesia, and can be used in conjunction with/or as an alternative to conventional preoperative oxygenation with a facemask.
- Extubation should be performed when the patient is close to fully awake and without residual sedation or neuromuscular weakness.
- Monitoring the patient in the PACU for recurrent respiratory events, including apnea, bradypnea, pain–sedation mismatch, CO_2_ retention, or oxygen desaturation can identify patients at high risk for postoperative respiratory complications and need for increased postoperative monitoring.
- Opioid analgesia, if possible, should be avoided; otherwise, it should be titrated slowly with careful monitoring.
- Caution should be advised in using high-flow supplemental oxygen in postoperative obese patients with known or suspected OHS, especially when they are sedated and on intravenous opioids.

Abbreviations: ABG, arterial blood gas; OHS, obesity hypoventilation syndrome; OSA, obstructive sleep apnea; PACU, post-anesthesia care unit (with permission from (https://journals.lww.com/anesthesia-analgesia/fulltext/2021/05000/obesity_and_obesity_hypoventilation,_sleep.13.aspx, accessed on 21 August 2024)).

## Abbreviations

BMI	Body Mass Index
AHI	Apnea–Hypopnea Index
OSA	Obstructive Sleep Apnea
SRH	Sleep-Related Hypoventilation
COPD	Chronic Obstructive Pulmonary Disease
OHS	Obesity Hypoventilation Syndrome
PSG	Polysomnography
ABG	Arterial Blood Gas
PH	Pulmonary Hypertension
FVC	Forced Vital Capacity
PFT	Pulmonary Function Test
FRC	Functional Residual Capacity
ERV	Expiratory Reserve Volume
ICU	Intensive Care Unit
CPAP	Continuous Positive Airway Pressure
LBW	Lean Body Weight
TBW	Total Body Weight
PEEP	Positive End Expiratory Pressure
CHF	Congestive Heart Failure
CAD	Coronary Artery Disease
NCS	Non-Cardiac Surgery
HELP	Head Elevation Laryngoscopy Position
PAP	Positive Airway Pressure
IPAP	Inspiratory Positive Airway Pressure
EPAP	Expiratory Positive Airway Pressure
TV	Tidal Volume
REM	Rapid Eye movement
PACU	Post-Anesthesia Care Unit
OIRD	Opioid-Induced Respiratory Depression
